# Improving the Assessment of Mild Cognitive Impairment in Advanced Age With a Novel Multi-Feature Automated Speech and Language Analysis of Verbal Fluency

**DOI:** 10.3389/fpsyg.2020.00535

**Published:** 2020-04-09

**Authors:** Liu Chen, Meysam Asgari, Robert Gale, Katherine Wild, Hiroko Dodge, Jeffrey Kaye

**Affiliations:** ^1^Center for Spoken Language Understanding, Oregon Health & Science University (OHSU), Portland, OR, United States; ^2^Department of Neurology, NIA-Layton Aging and Alzheimer's Disease Center, Oregon Health & Science University (OHSU), Portland, OR, United States; ^3^Department of Neurology, Oregon Center for Aging & Technology (ORCATECH), Oregon Health & Science University (OHSU), Portland, OR, United States; ^4^Department of Neurology, Michigan Alzheimer's Disease Center, University of Michigan, Ann Arbor, MI, United States

**Keywords:** neuropsychological tests, short term memory, animal fluency, biomarkers, mild cognitive impairment (MCI), computerized assessment

## Abstract

**Introduction:** Clinically relevant information can go uncaptured in the conventional scoring of a verbal fluency test. We hypothesize that characterizing the temporal aspects of the response through a set of time related measures will be useful in distinguishing those with MCI from cognitively intact controls.

**Methods:** Audio recordings of an animal fluency test administered to 70 demographically matched older adults (mean age 90.4 years), 28 with mild cognitive impairment (MCI) and 42 cognitively intact (CI) were professionally transcribed and fed into an automatic speech recognition (ASR) system to estimate the start time of each recalled word in the response. Next, we semantically cluster participant generated animal names and through a novel set of time-based measures, we characterize the semantic search strategy of subjects in retrieving words from animal name clusters. This set of time-based features along with standard count-based features (e.g., number of correctly retrieved animal names) were then used in a machine learning algorithm trained for distinguishing those with MCI from CI controls.

**Results:** The combination of both count-based and time-based features, automatically derived from the test response, achieved 77% on AUC-ROC of the support vector machine (SVM) classifier, outperforming the model trained only on the raw test score (AUC, 65%), and well above the chance model (AUC, 50%).

**Conclusion:** This approach supports the value of introducing time-based measures to the assessment of verbal fluency in the context of this generative task differentiating subjects with MCI from those with intact cognition.

## 1. Introduction

Early detection of the signs of transition from normal cognitive aging to Mild Cognitive Impairment (MCI) is highly valuable, particularly for preventing the transition to the more severe stages of dementia such as Alzheimer's disease (AD). Typically, quantitative assessment of cognition is carried out in the clinic by a trained psychometrician or neuropsychologist through a battery of cognitive tests that examine various aspects of cognitive abilities such as attention, memory, reasoning, and language skills (Borson et al., 2006; Woodford and George, 2007). Full conventional assessment, involving the use of pencil and paper, is a time-consuming process that can take up to several hours and may become burdensome when, for example, repeated re-assessment is required to monitor the progression of dementia. Not surprisingly, there has been a growing interest in developing more automated alternative methods that allow low-cost, scalable, and home-based cognitive assessments for detection of cognitive decline. More efficient methods of assessment can play a crucial role in screening for the detection of cognitive decline and can potentially target a broader segment of the population at more frequent intervals.

Among cognitive tests, verbal fluency (VF) tests have been widely used in several dementia screening batteries. In a VF test, participants are asked to name as many words in a category (e.g., animals) as possible in a short duration of time, typically 1 min. The VF test is administered in two different ways: (1) *semantic fluency*, in which participants are asked to generate words from a semantic category such as animals, fruits, or vegetables, and (2) *phonemic fluency* where participants must generate words that begin with a particular letter such as “F” or “S.” In the conventional scoring of VF tests, the count of uniquely generated words in the test comprises the final score. Prior research suggests that verbal fluency is a function of individuals' age regardless of cognitive functioning and younger populations perform better in this test compared to older adults (Alenius et al., 2019; Taler et al., 2019). Within older adults with normal cognition, Farina et al. (2019) highlights that within a short period of one's life, the rate of decline in VF score is not significant. In contrast, individuals with MCI achieve lower VF score than the healthy population and their score declines faster in the same period of time.

One disadvantage of conventional scoring is that it does not consider other clinically relevant information that can be captured from the response, such as the sequential pattern of words produced in a semantic fluency test (Taler et al., 2019). In an attempt at a more detailed assessment of an animal fluency (AF) test, Troyer et al. (1997) proposed a computational approach for characterizing the semantic retrieval process that revolves around two sub-processes (known as two-part memory retrieval): (1) *clustering*, in which a participant retrieves words that share some subcategories (e.g., *{dog, cat}* are both mammals), and (2) *switching*, in which a participant switches to a different semantic subcategory for retrieving a new word (e.g., *{[cat, falcon], [elephant, shark]}*) (Troyer et al., 1997). To quantify the semantic similarity of animal names for switching and clustering, they first manually developed a structured table that categorizes 545 animal names into 22 subcategories that cover a particular cluster of related animals (e.g., domestic animal, birds, etc.). Animal names in this table are not exclusive to a single subcategory and can be part of up to four subcategories. Next, they segmented each subject's answer into multiple clusters based on a semantic search model defined by them. Then, they counted the number of clusters and switches. Their experimental results using these measures show that young participants generate more words and also switch more frequently as compared to older participants. In another study, using count-based measures extracted from switching and clustering components of AF test (mean cluster size and count of switches), Troyer et al. (1998) experimentally verified the effectiveness of these two features for discriminating a group of patients with dementia from demographically matched control groups. We use this method of identifying *switches* and *clusters* in our experiments and refer to it as “Troyer.” Despite the potential usefulness of these features, there exist several limitations in computing switching- and clustering-based features from the preexisting manually constructed table of animal names. For example, the assignment of multiple subcategories to a word can cause an ambiguity in determination of subcategory switches (Woods et al., 2016). Alternatively, Woods et al. (2016) proposed a new computational method, explicit semantic analysis (ESA), based on the semantic relatedness of subsequent words computed in a vector space by cosine similarity distance given the *vector representation* of words. Unlike Troyer's approach that relies on a structured table of animal names, ESA detects the occurrence of a *switch* by comparing the pairwise cosine distance of two successive words to a predefined threshold value.

While count-based measures, derived from clustering and switching components, are powerful in capturing the semantic pattern of retrieved words, they fail to quantify the difficulty of retrieving a new word from a semantic cluster, often known as lexicon search strategy. Inspired by the *marginal value theorem (MVT)* (Charnov, 1976), Hills et al. (2012) proposed a computational method for characterizing the lexicon search strategy observed in individuals' AF test responses. Traditionally employed to model the foraging behavior of animals, MTV optimizes the *benefit-cost ratio*, the estimation of whether it is more beneficial to continue searching for food at the current patch of food vs. expending the effort to move some distance with the hope of discovering a more bountiful patch. This optimization problem in animals' foraging strategy resembles the semantic-retrieval strategy in the AF test in which one may retrieve more words over the course of the test if optimally choosing when to *switch* to a new *cluster*. The critical measures that allow for extracting higher level timing information from *switch* and *cluster* components (e.g., average time spent in clusters) which characterize the lexicon search strategy are *timestamps*—the start and end time of each recalled word in the response. Obtaining timestamps through manual annotation of verbal responses is a time-consuming process and also prone to subjective judgment. To mitigate this problem, Hills et al. (2012) visually presented the test instruction through a computer screen and asked participants to type in their retrieved animal names. This process allowed them to collect the timing of entries and to subsequently extract timing information. However, the nature of data acquisition in this proposed test administration framework assumes typing skills, which can be particularly problematic for those older individuals who may have physical conditions or might be less familiar with these devices.

In this study, we address this problem by introducing a computational method using an automatic speech recognition (ASR) system that automatically estimates the timestamps from the responses. Using the timestamps and identified *switch* and *cluster* components, we then extract a set of timing features following the approach proposed by Hills et al. (2012). Finally, we summarize the assessment of a response by augmenting its count-based features with its automatically extracted timing features. Focusing on the verbal responses of 70 subjects (28 with MCI and 42 demographically matched normal controls) who have performed an AF test, our ultimate goal is to develop automated machine learning algorithms for distinguishing MCI subjects from normal controls based on the combination of count-based and time-based features.

## 2. Materials and Methods

### 2.1. Data Collection and Corpus

The subjects in this study come from existing community cohort studies of brain aging at the Layton Aging & Alzheimer's Disease Center, an NIA-funded Alzheimer's center for research at Oregon Health & Science University (OHSU). The Clinical Dementia Rating (CDR) scale (Morris et al., 1997) was used, as a clinical reference, for classifying subjects into groups: CDR = 0.5, considered MCI, while CDR = 0, defined cognitively intact participants.

Individual audio recordings of 98 animal fluency test sessions along with the manual transcriptions of their verbal responses were used in study. Out of 98 participants, 28 were diagnosed with MCI and the remaining 70 participants were cognitively intact (CI). Our statistical analysis using the Student's *t*-test showed that there was a significant difference in the demographic factors of participants between MCI and CI groups. It is possible that observed changes in spoken language patterns of participants with MCI is the consequence of subject differences in demographic factors such as age or education level regardless of cognitive decline (Mathuranath et al., 2003). In order to control for demographic factors, we used a freely available package, “ldamatch,” that selects a subset of CI group which is statistically matched to the MCI group using exhaustive search (Gorman, 2016). Out of these 70 participants, Within the matched samples, all participants are White people except a participant from the CI group with Asian ethnicity. Table 1 reports the baseline characteristics of 70 sub-sampled participants (28 with MCI and 42 CI) of more equal educational level, age, and sex. Additionally, Mini-Mental State Examination (MMSE) (Cockrell and Folstein, 2002) and AF test scores (i.e., the total count of correctly retrieved animal names) are presented in this table.

**Table 1:** Baseline characteristics of MCI and demographically controlled participants.

**Variable**	**Intact**	**MCI**	***p*-value**
	***n* = 42**	***n* = 28**	
Age	89.9 (5.55)	91.2 (5.17)	0.32
Gender (% Women)	64.3%	50%	0.85
Years of Education	14.3 (2.70)	14.8 (2.79)	0.53
MMSE	28.0 (1.63)	26.0 (3.16)	0.08
AF score	17.3 (4.99)	13.3 (4.12)	0.04

*The Kolmogorov–Smirnov test was used to calculate p-values*.

### 2.2. Semantic Clustering

**Figure 1:**
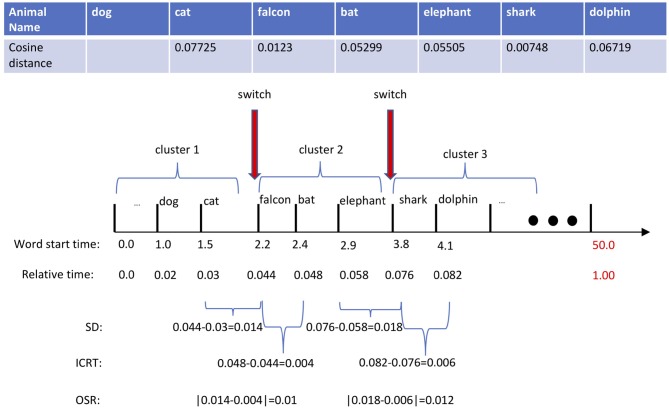
In this example, we manually set the threshold to 0.05. Based on that, there exists two switching positions marked by red arrows. The *switching duration (SD)* of the first switching is 0.7 | the time difference between falcon and cat. The *intra-cluster retrieval time (ICRT)* of the first switching is 0.2 | time difference between bat and falcon. Thus, the absolute difference between the *SD* and *ICRT* in the first switch, *optimal switching rate (OSR)*, will be 0.5.

As described above, the core of our computational method for characterizing verbal responses revolves around the *switch* and *cluster* components; from them, we subsequently extract two sets of count-based and time-based features. In our approach, we employ both methods previously mentioned: *Troyes-based* (Troyer et al., 1998) and *ESA-based* (Woods et al., 2016) methods. The difference between these methods lies in the semantic representation of animal names. Troyer's method uses a manually crafted table of animal names categorized in 22 semantically related subcategories: two animal names are semantically similar if they both belong to the same subcategories. While Troyer et al. (1997) represents an animal name precisely with an index of a subcategory, ESA represents it through a long vector in a high-dimensional vector space, in which semantically related words cluster around each other. Thus, using a similarity measure, such as cosine distance, one can readily identify how semantically close two words are. For more detail on ESA, we refer readers to Gabrilovich and Markovitch (2007). The top table in Figure 1 displays a partial response of a subject to the AF test, in which a sequence of produced words *{cat, falcon, bat, elephant, shark, dolphin}* is corresponded to the sequence of cosine similarities *d*(*W*_*i*−1_, *W*_*i*_) between the pair of previous and current words: *{–, 0.077, 0.012, 0.053, 0.055, 0.007, 0.067}*. According to ESA, the higher the cosine similarity, the stronger the relationship between the pair of two words. Practically, the cosine distance of two words is compared to a predefined threshold value—a critical factor in the success of the ESA method. In our working example, given the threshold value of 0.05, the word pair of *cat* and *falcon* is semantically different because *d*(*cat, falcon*) = 0.01 is lower than the threshold, 0.05. While typically a single threshold is used across all subjects, we adapt the threshold value to every subject following an approach proposed in Woods et al. (2016). For every subject, we first normalize the all cosine distances computed across all word pairs of the response to a unit range and 75% of the mean cosine distance is set as a subject-specific semantic threshold.

### 2.3. Computational Methods for Characterizing Verbal Responses

#### 2.3.1. Count-Based Features

After removing all non-animal words from a response, the following set of count-based features are extracted from the *switches* and *clusters* components: (1) the total number of unique animal words (standard AF score), (2) the total number of switches (NS), and (3) the average number of words in clusters (ANWC), (4) the total number of unique words, (5) the total number of duplicate words, (6) the mean of log of word frequency, (7) the standard deviation of word frequency, (8) the mean of words' syllables, (9) the standard deviation of words' syllables, (10) the mean of words' typicality, (11) the standard deviation of words' typicality, (12) the mean of ESA of adjoining words (MESA), (13) the mean of ESA between every word and every other word in the answer (MAESA), (14) the ratio between MESA and MAESA, (15) the total number of switches, (16) the average number of words in clusters, and (17) the total number of single word clusters. These features were extracted based on Woods et al. (2016)'s open-sourced code. Using a feature selection method (will be described at section 2.3.3.1), we ranked the relative importance of the these features and picked up the first three features as most informative features in our computational model. We also noticed numerous cases in our dataset where a single word appeared in its own cluster. To capture this phenomenon, we developed a new feature that measures the ratio between the count of single word cluster to the total number of cluster and refer to it as *single cluster ratio* (SCR).

#### 2.3.2. Time-Based Features

Our approach for characterizing the lexicon search strategy is based on the *marginal value theorem (MVT)* (Charnov, 1976), where we hypothesize that healthy individuals *optimally* switch to a new cluster leading to the production of more animal names while those individuals with MCI are less capable of finding optimum transition points. With a poor switching strategy, a participant either lingers too long in a cluster or moves too fast to a new cluster; ultimately producing fewer animal names. Based on this assumption, we define the following time-based features to capture the difficulty of retrieving a new word in an AF test: (1) *switching duration (SD)*, the elapsed time of the transition from one to another, and (2) *intra-cluster retrieval time (ICRT)*, the duration between the first two retrieved words in a new semantic cluster. The latter measures how fast one produces a new word once switched to a new cluster. Similar to Hills et al. (2012)'s approach, the central lower-level features required in computing SD and ICRT are timestamps. Along with the sequence of words, Figure 1 shows the sequence of timestamps *{1.0, 1.5, 2.2, 2.4, 2.9, 3.8, 4.2}* representing the onset of each word measured in seconds. Extracting the timestamps along with the ESA-derived *switches* and *clusters*, one can compute these features from our example as follows:

(1) $$SD_{1,2}=\{(t_{falcon}-t_{cat}),(t_{shark}-t_{elephant})\;=\{0.7,0.9\}$$

(2) $$ICRT_{1,2}=\{(t_{bat}-t_{falcon}),(t_{dolphin}-t_{shark})\;=\{0.2,0.4\}$$

Note that the proposed method for measuring ICRT remains valid if subjects were able to produce at least two animal words once switched to a new cluster. Next, we use SD and ICRT features and craft another feature, *optimal switch rate* (OSR), that estimates the success of a switch by measuring the difference between the switching duration and intra-cluster retrieval time as follows:

(3) $$\begin{array}{lll} OSR_{1,2} & = & \left\{\left|SD_{1}-ICRT_{1}|,|SD_{2}-ICRT_{2}\right|\right\}=\left\{0.5,0.6\right\} \end{array}$$

where |()| is the absolute value operator. The more successful a switch, the smaller the OSR. As the number of *switches* and *clusters* may vary across responses, the length of the SD and ICRT feature vectors vary accordingly. Noting that our ultimate objective is to utilize these features for learning classification algorithms, computed time-based features need to be summarized into a global feature vector of a fixed dimension for each read response. To unify the dimension of the global feature vector across all responses, each feature is summarized in terms of standard statistical aggregates such as mean, median, variance, minimum and maximum. In practice, this proposed computational approach will face a few limitations to be addressed in section 2.3.2.2.

##### 2.3.2.1. Time alignment

Manual extraction of timestamps from a response is a labor-intensive task and also prone to subjective judgment. To automate the process, we use the “forced alignment” algorithm implemented in the Kaldi ASR toolkit (Povey et al., 2011) to extract time information from a verbal response. Forced alignment is a sequence matching process in which a spoken audio segment is time aligned to a given sequence of words. Given the audio segment of an utterance and its corresponding word-level transcription, forced alignment automatically generates the word boundaries (e.g., the timestamps for when a word starts and ends in an utterance) from which we can compute subsequent higher-level features such as elapsed time between two successive words. For further details on “forced alignment,” we refer readers to McAuliffe et al. (2017).

##### 2.3.2.2. Practical issues

Ideally, a given response would consist of only animal names, but in reality, the recordings often contain extraneous speech, including filler and conversational words as well as interruptions from the examiner. Sometimes a participant will ask how much time remains for the test, and other times the examiner offers words of encouragement. In more than half of the recordings, both the examiner and the participant engaged in a bit of casual conversational speech, and the duration and frequency of such interruptions varied across the recordings. Encouragement from the examiner is usually a few short words (e.g., “good”), while a response to a time check might be a whole sentence (e.g., “you still have ten seconds left”). Prior to feature extraction, we remove all the non-animal words from the response. From a computational point of view, trimming out of these extraneous words results in a shortened response with altered timestamps that will ultimately influence the uniformity of time-based features across different subjects. To compensate for this issue, we first normalize the timestamps to the length of “shortened” audio and then measure the SD and ICRT features. In our working example shown in Figure 1, the original 58-s long recording was “shortened” to 50 s after filtering out non-animal words. Then, the *relative timestamps* are used for computing SD and ICRT features. Figure 1 shows both SD and ICRT features before and after time normalization.

Another common issue in the AF test is the presentation of plural forms of animal names (e.g., “pigs” rather than “pig”). In order to normalize all animal names in transcriptions to their singular forms, we apply a word stemmer algorithm on responses to remove morphological affixes from words, leaving only the word stem. Also note that many animal names follow irregular pluralization rules (i.e., “hippopotami” and “oxen”). Additionally, some animal names contain more than one word (i.e., “great white shark” and “mountain lion”). To tackle this problem, we built a tool to retrieve the normalized form from the internet. First, we found the singular form of each noun from the Merriam-Webster website, then we referenced Wikipedia to decide whether a multi-word sequence is an animal name. Conveniently, the Wikipedia template for an article about a species includes a biological taxonomy table, so upon looking up a given species, we could confirm it belongs to the kingdom Animalia to positively identify an animal name.

#### 2.3.3. Machine Learning Algorithms

**Figure 2:**
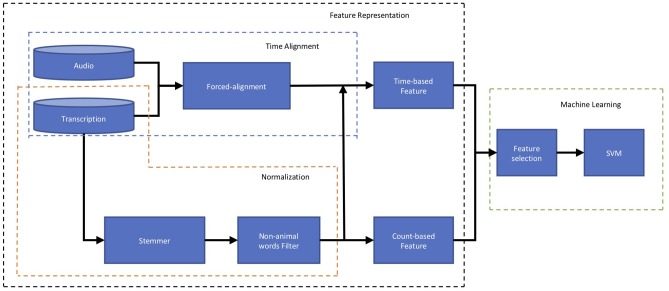
Block diagram of computational framework including to distinguish participants with MCI from those with intact cognition based on Audio recording and Transcription of their responses to an animal fluency test. The first block of this plot, *Feature Representation* (shown by the black box), represents the characteristics of the response using *Time-based* and *Count-based* features. The second block, *Machine Learning* (shown by the green box), first picks up the more informative features through the *Feature Selection* and using those, predicts the participant's cognition status (MCI or intact).

Figure 2 outlines the flow of data that produces time- and count-based features from verbal responses to distinguish participants with MCI from those with intact cognition. Given an audio recording and a word-level transcription for a verbal response, we extract both count-based features and time-based features and augment them into a global feature vector. As noted earlier, time-based features are derived from the timestamps which are automatically extracted by the forced-alignment algorithm described in section 2.3.2.1. Representing a verbal response as a global feature vector allows for training a classification algorithm, support vector machine (SVM) (Smola and Schölkopf, 2004) classifier. A SVM classifier is a discriminative model that attempts to distinguish between two classes of data points separated by a hyperplane in a high dimensional space. The parameters of the hyperplane are learned from a set of training examples. We trained linear and non-linear SVM classifiers employed from the open-source Scikit-learn toolkit (Pedregosa et al., 2011). All experimental results, presented in the next sections, are based on the linear SVM as it outperformed the non-linear SVM. We also repeated the experiment using a “Chance” classifier which randomly assigned participants into MCI and CI classes.

Prior to training a SVM model, we scale the range of computed features into a constrained range using Scikit-learn's *RobustScaler*. This is a necessary step in our computational framework as we noticed that the range of derived features greatly differ from each other. For example, the number of *correct animal words* ranges from 4 to 30 within the responses while the *mean count of switches* ranges from 2 to 9. Features with large scale will dramatically impact what the machine learning algorithm would learn and erase most benefit that features with smaller scale offers. *RobustScaler* centers and scales the data according to the following equation, operating separately on each dimension of the global feature vector:

(4) $$\begin{array}{l} f\left(x_{i}\right)=\frac{x_{i}-Q_{1}\left(x\right)}{Q_{3}\left(x\right)-Q_{1}\left(x\right)} \end{array}$$

where *x*_*i*_ denotes to the *ith* feature; *Q*_1_(***x***) and *Q*_3_(***x***) are the feature's 25*th* and 75*th* quantiles, respectively.

##### 2.3.3.1. Feature selection

Employing a large number of features to train a statistical model, such as an SVM classifier, may lead to overfitting and result in lack of generalizability. It has been previously shown that a strategically reduced subset of features can significantly improve the model's predictive power (Hastie et al., 2005). From the Scikit-learn toolkit (Pedregosa et al., 2011), we chose a feature selection technique known as *recursive feature elimination with cross-validation* (RFECV), which ranks the importance of features based on a given scoring function and returns a subset of features. It constructs a smaller subset of features, and calculates the model performance given each remaining subset. The elimination process continues until all features are exhausted. Finally, the feature set that maximizes the model performance across all feature sets is selected as the best performing feature set.

#### 2.3.4. Performance Criteria

To evaluate the performance of the proposed classifier, we adopted the following evaluation metrics: (1) Sensitivity - the portion of correctly identified MCI participants (true positives). Sensitivity assesses the capability of the model to distinguish MCI from cognitively intact participants; (2) Specificity - the portion of correctly identified cognitively intact participants (true negative). Specificity measures how well the model avoids false positives; and (3) Area under the curve of receiver operating characteristics (AUC ROC). The most common method for evaluating the performance of a binary classifier is the ROC (Hanley and McNeil, 1982), which plots the *sensitivity* (true positive rate) of the classifier vs. *1-specificity* (false positive rate) of the classifier as the classification threshold varies. We use a classification threshold in a grid search schema to cover the most positive threshold (everything true) to the most negative threshold (everything false).

#### 2.3.5. Cross-Validation on the Imbalanced Dataset

To demonstrate whether our statistical analyses and experimental results were independent of our data sets, we used cross-validation (CV) techniques in which the train and test sets are rotated over the entire data set. In an imbalanced dataset, a machine learning algorithm receives more information from the class which has more samples and consequently may not learn properly from the smaller-sized class. To overcome this problem in our imbalanced dataset, we use a special leave-one-pair-out (LOPO) cross validation scheme. LOPO cross validation first slices the dataset into multiple pairs, each includes one CI instance and one MCI instance. At every iteration, it selects one pair as testing set and from the rest, creates a balanced training set by randomly selecting instances from the class with larger samples. In our example with 28 MCI and 42 CI samples, leaving one pair for the test, LOPO randomly selects 27 CI out of remaining 41 CI samples at each training iteration. To reduce the effect of randomization in our final results, we shuffle the data and repeat the LOPO cross validation 500 times. Lastly, we average across 500 final scores and report that as the performance of our model.

## 3. Results

### 3.1. Statistical Analysis

**Figure 3:**
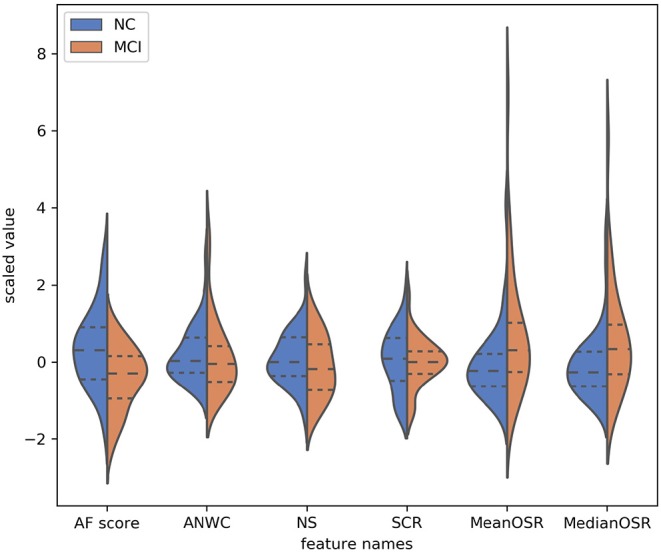
Probability distribution of features (y-axis) selected by the feature selection algorithm. The dynamic range of features have been normalized according to the *RobustScaler* approach. The dotted line from left to right are 25% quantile, 50% quantile, and 75% quantile.

To explore the effectiveness of our proposed features in differentiating subjects with MCI from CI controls, we conducted a statistical analysis on extracted features using Kolmogorov-Smirnov test. As shown in Table 2, both *mean OSR* and *median OSR* features significantly distinguish the two groups with *p*-values of *p* < 0.04, *p* < 0.3, respectively. Probability distributions of the extracted features, depicted in Figure 3, show that the average of both *mean OSR* and *median OSR* features across CI controls is less than the average of those with MCI. This indicates that CI controls have less difficulty retrieving a new word upon a switch to a new cluster as compared to those with MCI. The statistical analysis also shows the discriminative power of the raw AF score for differentiating these two groups. The last two features picked up by our feature selection method, ANWC and the NS, were not able to detect a significant difference between MCI and CI groups.

**Table 2:** Kolmogorov–Smirnov test results of features that use ESA for semantic representation.

**Feature name**	**Statistic**	***p*-value**
AF score	0.33	0.04
ANWC	0.25	0.21
NS	0.21	0.39
SCR	0.13	0.91
MeanOSR	0.35	0.03
MedianOSR	0.36	0.02

### 3.2. Classification Results

We compared the performance of our trained SVM classifiers for distinguishing between participants with MCI and the CI controls. We independently extracted time and count based features from *switch* and *cluster* components identified by both Troyer's table-based and Troyer et al. (1997) and ESA semantic similarity based approaches to explore the effectiveness of these methods on extracted features. Not all the extracted features are expected to be useful, and in fact many are likely to be noisy. We applied the RFECV feature selection method, described at section 2.3.3.1, to extracted features and evaluated several models using cross-validation to pick the one with optimal performance. Ultimately, three count-based and three time-based features, presented in Table 2, were picked for the classification models. To understand the contribution of the different features, we introduced them incrementally and measured their performance across the LOPO cross-validations in terms of Sensitivity, Specificity, and AUC ROC reported in Table 3. For each SVM model in this table, we first optimized its hyper-parameters including the margin of the decision function, shown as “C” in the table, and of regularization form (either *L*_1_ or *L*_2_) that is added to the SVM cost function as an additive penalty term. The first SVM model was estimated with count-based features and subsequently, time-based features were introduced. To explore the effectiveness of features, we repeated the experiment and compared the results of our final models with three baseline models: a “Chance” classifier which randomly assigned participants into MCI and intact classes, and two SVM classifiers trained on demographic features of subjects (age, gender, and years of education and referred to as Dem.) and conventional AF score. As described earlier (section 2.1), subjects in both groups are demographically matched, and thus the performance of the SVM trained on demographic features is close to the “Chance” model. As shown by the results, all SVM classifiers outperform all three baseline models in terms of ROC AUC. Results also indicate that addition of time-based features—whether extracted based on ESA or Troyer methods—further improves the performance.

**Table 3:** Classification results using selected features (mean over 500 leave-pair-out spatial cross-validation repeats).

**Method**	**Features**	**ROC AUC (%)**	**Sensitivity (%)**	**Specificity (%)**	**SVM hyperparameter**
Troyer-Based	Count	70.25	62.71	66.48	*C* = 0.1, Penalty = *L*_2_
	Count + Time.	77.76	76.02	67.11	*C* = 10, Penalty = *L*_1_
ESA-Based	Count	73.81	75.46	60.46	*C* = 10, Penalty = *L*_1_
	Count + Time	77.09	70.27	69.68	*C* = 10, Penalty = *L*_1_
	Dem.	59.30	49.25	59.64	*C* = 10, Penalty = *L*_2_
	AF score	65.63	67.30	63.04	*C* = 1*10^−10^, Penalty = *L*_2_
	(Chance Model)	50.00	49.56	49.99	*C* = 1, Penalty = *L*_2_

### 3.3. Effectiveness of Correct Identification of Switch and Cluster Components

**Figure 4:**
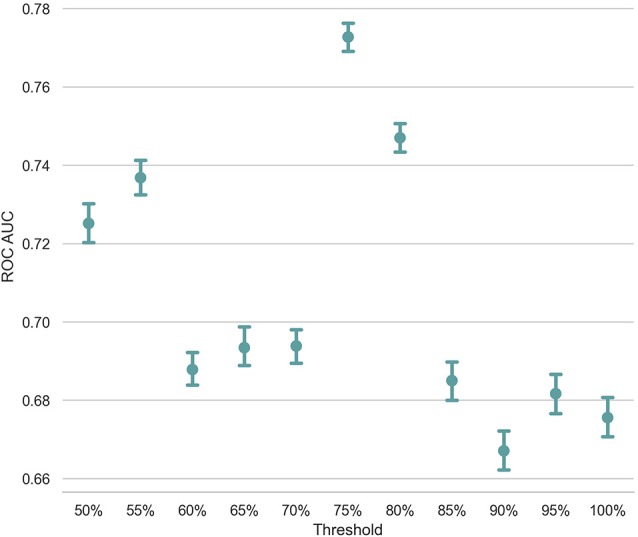
The x-axis is the different threshold setting (*xx%*) of mean cosine similarity of an individual's answer. The y-axis is the ROC AUC score.

Unlike Troyer's approach, in which a structured table of animal names identifies the switch and cluster components, the ESA-based approach uses a predefined threshold for deciding whether two consecutive words belong to the same semantic cluster. For example, in Figure 1, we set the threshold to be 0.05 for this subject and segment these seven animal names into three clusters based on the threshold. If we change the threshold and set it to be 0.06, *cluster2* will be split into three clusters such that each cluster only contains one term. Since the discriminative power of count-based and time-based features highly depends on the identification of switch and cluster components in the response, one question is how the threshold setting influences the classification result. In order to gauge the influence of this factor, we incrementally increased the threshold from 50 to 100% with a step size of 5% and created 11 feature sets. Using these distinct feature sets extracted from all subjects except a pair of randomly selected subject purposely left for the test, we trained 11 SVM models and measured the AUC ROC of the classification task on the pairs of test subjects. Figure 4 presents the average AUC ROC across 500 iterations of LOPO cross-validation at each threshold value. As shown in this figure, the classification model reaches to the highest AUC ROC with a threshold of 75%. This plot also verifies the importance of switch and cluster components, identified as a function of the threshold value, in the performance of the classification model.

## 4. Discussion

We have shown that a widely adopted form of a verbal fluency test (animal category fluency) used in cognitive assessment batteries distinguished MCI from demographically matched CI control participants (Cooper et al., 2004; Radanovic et al., 2009). Using count-based fluency scoring resulted in similar diagnostic category discrimination as reported by others using conventional counting (Oh et al., 2019). Others have used ASR techniques to examine the VF test. In Pakhomov et al. (2015), the same Kaldi ASR toolkit (Povey et al., 2011) and in König et al. (2018), Google's Automatic Speech Recognition (ASR) service were used for automatic transcription of responses. These studies either attempt to predict the raw VF score based on automatically generated response (Pakhomov et al., 2015) or only investigate count-based measures beside the raw VF score for differentiating MCI from cognitively intact participants (König et al., 2018). In contrast, the crux of work that differentiates it from these studies is how we employ the ASR system not only for automatic transcription but to perform the “forced alignment” algorithm for quantifying the temporal properties of verbal responses leading to the extraction of time-based measures. As showed by our experimental results, our time-based measures significantly improved the accuracy of our classification model.

In considering the goal of automating the administration and scoring of this test, we developed a method to go beyond conventional scoring that relies on the number of correctly produced category items. This unsupervised approach ultimately will require an algorithm that can be objectively applied employing machine learning to discern not only the simple counts, but other aspects that may add to the discriminatory power of the VF test. We experimentally showed that the conventional test score (i.e., the number of correctly recalled animal names within a minute) cannot capture other clinically useful information from the test and once it is solely used for training a SVM classifier, the resulting model achieved a poor performance. To mitigate this shortcoming, we proposed a computational approach for automatically analyzing the verbal responses via a set of time-based features that characterize the semantic search strategy during the word retrieval process. We statistically showed that these proposed features can differentiate individuals with MCI from CI controls. Additionally, they positively contributed toward the performance of a SVM classification model once they were added to standard count-based features. In spite of promising results achieved through the proposed computational model, considerable work remains to improve accuracy of the classification algorithms. Our analysis relied on animal names that were included in the Troyer et al. (1997)' table. However, there are always animal names that are unknown to this table and current analyses treat them as non-animal names and that impacts our assessment. A valuable avenue for future research would be to explore the feasibility of natural language processing (NLP) techniques to address this limitation using more sophisticated methods of word representation that is not limited to a word table. Addressing these limitations in future work is expected to result in viable speech-based outcome measures, derived from the verbal fluency test, for individuals with a range of neurodevelopmental disorders including MCI and Alzheimer's disease. The proposed methodology can increase the capacity for screening/detection of MCI by employing measures that cannot be easily computed manually in real time.

## Data Availability Statement

The raw data supporting the conclusions of this article will be made available by the authors, without undue reservation, to any qualified researcher.

## Ethics Statement

The studies involving human participants were reviewed and approved by The Oregon Health & Science University Institutional Review Board. The patients/participants provided their written informed consent to participate in this study.

## Author Contributions

All authors listed have made a substantial, direct and intellectual contribution to the work, and approved it for publication.

### Conflict of Interest

The authors declare that the research was conducted in the absence of any commercial or financial relationships that could be construed as a potential conflict of interest.
